# Association of Consecutive Influenza Vaccinations and Pneumonia: A Population-Based Case-Control Study

**DOI:** 10.3390/ijerph16061078

**Published:** 2019-03-26

**Authors:** Liang-Tsai Yeh, Chi-Ho Chan, Shun-Fa Yang, Han-Wei Yeh, Ying-Tung Yeh, Yu-Hsun Wang, Ming-Chih Chou, Chao-Bin Yeh, Ying-Hock Teng

**Affiliations:** 1 Institute of Medicine, Chung Shan Medical University, Taichung 40201, Taiwan; 68990@cch.org.tw (L.-T.Y.); ysf@csmu.edu.tw (S.-F.Y.); cshy236@csh.org.tw (M.-C.C.); sky5ff@gmail.com (C.-B.Y.); 2 Department of Anesthesiology, Changhua Christian Hospital, Changhua 50006, Taiwan; 3 Department of Microbiology and Immunology, Chung Shan Medical University, Taichung 40201, Taiwan; chiho@csmu.edu.tw; 4 Department of Medical Research, Chung Shan Medical University Hospital, Taichung 40201, Taiwan; cshe731@csh.org.tw; 5 School of Medicine, Chang Gung University, Taoyuan City 33302, Taiwan; george66889@gmail.com; 6 Graduate Institute of Oral Sciences, Chung Shan Medical University, Taichung 40201, Taiwan; yehtungtung@hotmail.com; 7 School of Dentistry, Chung Shan Medical University, Taichung 40201, Taiwan; 8 Department of Dentistry, Chung Shan Medical University Hospital, Taichung 40201, Taiwan; 9 Department of Emergency Medicine, School of Medicine, Chung Shan Medical University, Taichung 40201, Taiwan; 10 Department of Emergency Medicine, Chung Shan Medical University Hospital, Taichung 40201, Taiwan; 11 Department of Health Policy and Management, Chung Shan Medical University, Taichung 40201, Taiwan

**Keywords:** influenza vaccination, pneumonia, population-based case-control study

## Abstract

The purpose of this study was to investigate whether individuals receiving influenza vaccines have a lower risk of pneumonia. A nationwide population-based case-control study was conducted using data from the National Health Insurance Research Database in Taiwan. We enrolled 7565 patients each in pneumonia and non-pneumonia groups after diagnosis of patients with chronic pulmonary disease, and these patients were individually age and sex matched in a 1:1 ratio. Using conditional logistic regression analysis, adjusted odds ratios (aORs) were estimated in patients who received influenza vaccination and those who had not previously had pneumonia. Moreover, we also analyzed the interval between vaccination and the onset of pneumonia and the number of vaccinations received by patients. This was compared with patients who never received influenza vaccination. Patients who had received influenza vaccination and had been vaccinated for two consecutive years (aOR = 0.85, confidence interval (CI) = 0.79–0.93 and aOR = 0.75, CI = 0.67–0.85, respectively) showed lower rates of pneumonia occurrence by 15–25%. In conclusion, influenza vaccination significantly reduces the occurrence of pneumonia, especially in individuals who receive vaccination in consecutive years.

## 1. Introduction

Pneumonia is a common clinical presentation after a respiratory infection. Since 2016, pneumonia has been the third most common cause of death in Taiwan. The majority of pneumonia cases can be classified as either community-acquired, hospital-acquired, or acquired after traveling to foreign countries. Bacterial pneumonia is usually a complication of influenza virus infection [1]. The American Thoracic Society and Infectious Diseases Society of America have classified pneumonia into three types, namely community-acquired pneumonia (CAP), hospital-acquired (or nosocomial) pneumonia, and ventilator-associated pneumonia, according to the epidemiology, pathogenesis, and risk factors for infection in patients with pneumonia [2,3]. The etiology of pneumonia includes bacteria, viruses, fungi, and protozoa. Generally, pathogens that potentially cause pneumonia subsist of “typical” bacteria and “atypical” organisms, including *Mycoplasma pneumoniae*, and respiratory viruses, such as influenza viruses. However, 11–20% of pneumonia cases are polymicrobial, and the etiology usually consists of a combination of typical and atypical pathogens [4]. *Streptococcus pneumoniae* has been reported as the major pathogen in secondary infection after individuals were infected by influenza viruses, and it increases the mortality risk of patients [5,6,7,8]. Previous studies such as Tessmer et al. [9] show that prior influenza vaccination is associated with a less severe clinical course and improved long-term survival in patients with CAP, especially during the influenza epidemic season. However, Shinjoh et al. [10] reported that children who were immunized for two consecutive seasons experienced decreased vaccine effectiveness and were more likely to acquire influenza and that this might be associated with immunity against influenza infection in the previous season. Therefore, the aims of this study are also to clarify this.

Among the pathogens, influenza is one of the primary causes of pneumonia and influential respiratory diseases [11,12]. In general, human influenza viruses include A/H1N1, A/H3N2, and B viruses. Because of the antigenic drift of viruses, epidemics of influenza are reported every year in Taiwan. To effectively prevent the disease, large influenza vaccination programs have been held by the health authority of the Taiwanese government, and the trivalent influenza vaccine containing influenza A/H1N1, A/H3N2, and B viruses is employed annually. To increase the influenza vaccination coverage, free-of-charge vaccination programs have been held since 1988 for several groups, including young children under 6 months old, students from primary school to senior high school, and adults with high influenza risk (i.e., people aged over 50 years and patients of any age with a chronic illness like diabetes, chronic hepatitis, cardiovascular disease, chronic pulmonary disease, chronic renal disease, etc.) [13]. In fact, a characteristic of influenza infection in the elderly is a high frequency of pneumonia complications. Therefore, inoculation with the influenza vaccine is critical for both preventing influenza infection and lowering the risk of post-influenza pneumonia development in the elderly [14]. Influenza vaccination has been reported to be associated not only with prevention of influenza epidemics but also reduced risk of several diseases, such as acute kidney injury, diabetes, cardiovascular disease, and respiratory failure in chronic obstructive pulmonary disease, especially in elderly people [15,16,17,18,19,20]. However, the relationship between pneumonia and influenza vaccination remains unclear. This study investigated whether individuals receiving influenza vaccines have decreased risk of developing pneumonia.

## 2. Materials and Methods

### 2.1. Data Source

We used the National Health Insurance Research Database (NHIRD) of Taiwan from 1 January 2009 to 31 December 2013. The NHIRD contains all medical data, including information on disease diagnosis, drug prescriptions, medical operations, and medical expenses of insurance claims data. The National Health Insurance covered more than 99% of Taiwan’s population by 2010. The Longitudinal Health Insurance Database 2010 included data from 1 million people randomly sampled from the NHIRD and who were registered by the end of 2010. Diagnostic codes were recorded according to the International Classification of Diseases, Ninth Edition, Clinical Modification (ICD-9-CM). This study was approved by the ethical review board of Chung Shan Medical University Hospital (CSMU No. CS 18096).

### 2.2. Study Groups

This study used a case-control study design. All subjects had been diagnosed with chronic pulmonary diseases (ICD-9-CM 490–496) from 2010 to 2012. To confirm the accuracy of diagnosis, we enrolled patients with a minimum of two outpatient visits or one admission. The case group was defined as newly diagnosed pneumonia (ICD-9-CM 481, 482, 483, 485, and 486) from emergency or hospitalization after chronic pulmonary disease diagnosis. The first date of pneumonia was the index date. Patients with no pneumonia diagnosis from 2009 to 2013 were selected as the control group. We performed a 1:1 age and sex match to obtain index dates corresponding to the control group.

### 2.3. Exposure Measurement

Influenza vaccination was defined using an ICD-9-CM (V04.7 or V04.8) diagnosis or a drug code within two years before the index date. Comorbidities considered were hypertension (ICD-9-CM 401–405), diabetes mellitus (ICD-9-CM 250), cerebrovascular disease (ICD-9-CM 430–438), renal disease (ICD-9-CM 582–582.9, 583–583.7, 585, 586, and 588–588.9), liver disease (ICD-9-CM 456.0–456.21, 571.2, 571.4–571.49, 571.5, 571.6, and 572.2–572.8), ischemic heart disease (ICD-9-CM 410–414), dementia (ICD-9-CM 290.0–290.4, 294.1, 331.0–331.2, and 331.82), alcohol-related disorder (ICD-9-CM 291, 303, 305.0, 571.0, 571.1, 571.3, 790.3, and V11.3), and malignancy (ICD-9-CM 140–208). All patients were diagnosed with the disease before the index date.

### 2.4. Statistical Analysis

A comparison of pneumonia and non-pneumonia groups was performed using a Chi-squared test or independent *t*-test, as was appropriate. The association between influenza vaccination and pneumonia was analyzed using conditional logistical regression analysis. The statistical software used was SPSS V.18.0 (SPSS, Chicago, IL, USA), and the significance was defined as *p* < 0.05. On the use of post hoc power analysis of the logistic regression, the alpha error was 0.05 and the beta error was 0.2. The achieved power of the study was 0.83.

## 3. Results

**Figure 1:**
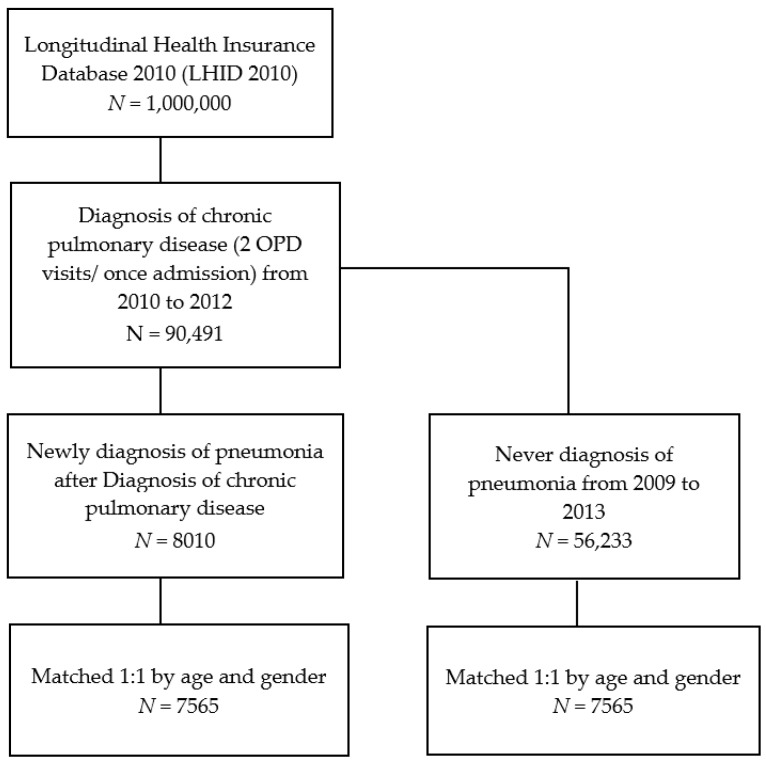
Flow chart for patient selection.

In total, 90,491 patients diagnosed with chronic pulmonary disease were initially recruited from 2010 to 2012. Next, 8010 of the 90,491 patients were selected according to a new diagnosis of pneumonia after chronic pulmonary disease diagnosis. Another 56,233 patients who were never diagnosed with pneumonia were also selected. After this, we performed a 1:1 age and sex match, and 7565 patients were enrolled in each of the two groups: pneumonia and non-pneumonia (Figure 1). The distributions of age and sex in the groups were not significant (Table 1).

Next, we analyzed the risk of pneumonia and the selected comorbidities. Patients who had received an influenza vaccination showed a 15% decreased risk of pneumonia (adjusted odds ratio (aOR) = 0.85, confidence interval (CI) = 0.79–0.93). Furthermore, patients had an increased risk of pneumonia with following comorbidities: hypertension, diabetes, cerebrovascular disease, renal disease, and liver disease (Table 2).

We also analyzed the interval between the onset of pneumonia and receiving the influenza vaccination, as well as the number of vaccinations. We compared these results with patients who had never received an influenza vaccination. Patients that received the vaccine one year prior to the study showed a 13% reduction in the risk of developing pneumonia (aOR = 0.87, CI = 0.78–0.98). Patients vaccinated for two consecutive years prior to the study showed a 25% decreased risk of developing pneumonia (aOR = 0.75, CI = 0.67–0.85). Furthermore, patients vaccinated for three consecutive years compared with those who had never received influenza vaccination showed a 44% decreased risk of developing pneumonia (aOR = 0.56, CI = 0.45–0.69) (Table 3).

Finally, we performed a subgroup analysis by age and sex for the vaccination and non-vaccination groups. After we subdivided the patients by age into three subgroups (<40, 40–65, and ≥65), we observed that only those patients aged ≥ 65 had an obvious reduced risk of developing pneumonia (aOR = 0.78, CI = 0.71–0.86). Male patients also showed a lower risk of pneumonia than the female patients. However, vaccination did not reduce the risk of pneumonia in patients aged 40–65 years (Table 4).

## 4. Discussion

Patients diagnosed with chronic pulmonary disease were enrolled to analyze the association between influenza vaccination and the prevention of pneumonia. Our study indicated that patients who received vaccination one year prior to the study had a significantly decreased risk of developing pneumonia. Additionally, patients who received vaccination consecutively for two and three years showed a continuous reduction of their risk of pneumonia. Although the patients who received influenza vaccinations only in the second year did not show any decrease in their risk of developing pneumonia, the data was not statistically significant. Repeated influenza vaccination was demonstrated to be effective for preventing severe and fatal influenza infection in elderly individuals [17]. Our study showed that influenza vaccination can lower the risk of pneumonia in individuals aged ≥ 65. A previous study used health administrative databases to investigate the association between influenza vaccination and the all-cause death of elderly people >65 years. They also concluded that influenza vaccination was associated with reductions in the total hospitalizations for pneumonia and influenza and all-cause mortality during the influenza season [21]. Moreover, some studies, such as Li et al., confirmed that previous pneumococcal and influenza vaccination in elderly patients reduced the length of hospital stay and reduced the risk of bacteremia [22], which was similar to our study. However, Demirdogen Cetinoglu et al. showed that influenza vaccination did not affect the clinical outcome of hospitalized adult CAP patients. This result may be related to the low influenza vaccination rate in the elderly [23]. Therefore, further research is needed to analyze this in the future.

Several studies have shown that influenza vaccination not only prevents influenza infection but also reduces the risk of several diseases in certain groups of patients. According to a retrospective cohort study, the administration of the influenza vaccination in elderly patients with diabetes reduced risks of hospitalization, lung failure, and 12-month mortality [15]. Influenza vaccination in elderly individuals reduced the risk of acute kidney injury in a nested-control study. Although the actual mechanism is unclear, influenza vaccination is proposed to be associated with the reduction of the inflammation cascade [19]. A study showed that influenza vaccination reduced dementia risk in patients with chronic kidney disease [24]. Another study also showed that influenza vaccines might prevent cardiovascular disease [16]. A high-dose influenza vaccine (60 μg of hemagglutinin from each of the three viral strains against 15 μg of hemagglutinin from each of the four viral strains) might be more effective in reducing poor clinical outcomes in patients who have a heart failure or myocardial infarction history [20]. In another study, it was found that influenza infection increased the risk of atrial fibrillation, and people who received influenza vaccination showed a lower atrial fibrillation risk [18]. Influenza vaccination could decrease respiratory failure risk in patients with chronic obstructive pulmonary disease [13]. Our study found that influenza vaccination could significantly decrease the risk of pneumonia.

One of the most serious complications of influenza infection is bacterial pneumonia, which increases morbidity and mortality [25]. Evidence shows that older patients, or those with severe illness with pneumonia, had higher 30-day mortality rates [5]. In general, influenza viruses cause only the desquamation of the epithelial cells in the respiratory tract, but this disrupts the outermost part of the mucosal defense and promotes secondary bacterial pneumonia. The most common pathogens associated with secondary infection are *S. pneumoniae*, *Staphylococcus aureus*, and *Haemophilus influenzae* [26]. In Taiwan, to prevent *S. pneumoniae* infection in elderly individuals, the government has provided free vaccination of the 23-valent pneumococcal polysaccharide vaccine for people aged > 75 since 2008 [27]. We also considered that one of the factors for lowering pneumonia risk in our enrolled patients might be *S. pneumoniae* vaccination. However, only 257 (1.7%) patients in our study population had received *S. pneumoniae* vaccine. Therefore, the observation that influenza vaccination could reduce the risk of pneumonia in elderly individuals may not be overestimated.

The prevalent strains of influenza viruses in Taiwan are A/H1N1, A/H3N2, and B viruses. For the prevention of an influenza outbreak, the health authority of the Taiwanese government recommends trivalent influenza vaccine use. However, because of genetic divergence, influenza B viruses are divided into two lineages: Victoria and Yamagata lineages. Simultaneous cocirculation of these lineages has been observed, and a mismatch of the vaccine lineage and circulating strains has been reported [28,29,30]. This might decrease the effectiveness of influenza vaccines. However, there was no mismatch of the vaccine strains and the circulating strains in our study during the observation period (2010–2013).

Our study has several limitations. First, in Taiwan, influenza vaccines are purchased from different pharmaceutical companies. The manufacturing process of influenza vaccines varies between pharmaceutical companies. For instance, the inactivated influenza vaccine (i.e., Optaflu©) produced by Novartis contains whole virus, whereas the vaccine produced by Sanofi Pasteur (i.e., Fluzone©) contains split virus. We did not examine whether the protective effect of both these influenza vaccines is the same. In addition, the protective effect may be different for different age groups. Second, we were unable to obtain potentially relevant personal behavioral information, such as alcohol consumption, smoking habits, and body mass index. These confounding factors may have affected the outcome. Third, Taiwan’s National Health Insurance system involves the Taiwanese population. Our data accurately reflects the situation in Taiwan; however, our results may not be applicable to other regions. Fourth, the National Health Insurance Research Database (NHIRD) consists of claim data. The database used does not contain information about clinical parameters, such as pneumonia infection severity. Therefore, we could not further distinguish the severities of respiratory diseases. Fifth, the laboratory data and microbial culture data that may affect the occurrence of pneumonia infection are not included in the database. Moreover, information about from sputum cultures or viral swabs and the causative agents of pneumonia was not available. Thus, the cause of pneumonia identified could not be divided into viral or bacterial.

## 5. Conclusions

In conclusion, patients who receive influenza vaccination have a significantly lower risk of developing pneumonia, especially for the elderly (aged ≥ 65). The preventative effects against pneumonia depend on consecutive years of vaccination.

## Figures and Tables

**Table 1:** Demographic characteristics of pneumonia and non-pneumonia.

Variable	Pneumonia (*N* = 7565)		Non-Pneumonia (*N* = 7565)		*p*-Value
	*n*	%	*n*	%	
Vaccination					0.026 *
No	5006	66.2	4876	64.5	
Yes	2559	33.8	2689	35.5	
Age					1
<40	1739	23.0	1739	23.0	
40–65	1383	18.3	1383	18.3	
≥65	4443	58.7	4443	58.7	
Mean ± SD	59.1 ± 29.1		59.1 ± 29.1		1
Gender					1
Female	2991	39.5	2991	39.5	
Male	4574	60.5	4574	60.5	
Hypertension	3694	48.8	3173	41.9	<0.001 **
Diabetes	1673	22.1	1116	14.8	<0.001 **
Cerebrovascular disease	1425	18.8	693	9.2	<0.001 **
Renal disease	591	7.8	301	4.0	<0.001 **
Liver disease	385	5.1	298	3.9	0.001 **
Ischemic heart disease	1435	19.0	1111	14.7	<0.001 **
Dementia	665	8.8	262	3.5	<0.001 **
Alcohol-related disorder	58	0.8	15	0.2	<0.001 **
Malignancy	1009	13.3	406	5.4	<0.001 **

* *p* < 0.05, ** *p* < 0.01.

**Table 2:** Conditional logistic regression between influenza vaccination and comorbidities and the risk of pneumonia.

Variable	Crude OR	95% CI	Adjusted OR ^†^	95% CI
Vaccination				
No	1		1	
Yes	0.91	** 0.84–0.98	0.85	** 0.79–0.93
Hypertension	1.48	** 1.37–1.60	1.26	** 1.16–1.37
Diabetes	1.73	** 1.58–1.89	1.46	** 1.33–1.62
Cerebrovascular disease	2.48	** 2.24–2.76	2.15	** 1.92–2.41
Renal disease	2.06	** 1.78–2.38	1.64	** 1.41–1.92
Liver disease	1.31	** 1.12–1.54	1.11	0.94–1.32
Ischemic heart disease	1.40	** 1.28–1.53	1.35	** 1.22–1.49
Dementia	2.95	** 2.52–3.45	2.72	** 2.3–3.23
Alcohol-related disorder	4.07	** 2.27–7.31	3.68	** 1.99–6.82
Malignancy	2.88	** 2.54–3.27	3.10	** 2.71–3.55

^†^ Adjusted for hypertension, diabetes, cerebrovascular disease, renal disease, liver disease, ischemic heart disease, dementia, alcohol-related disorder, and malignancy. OR: odds ratio; CI: confidence interval; ** *p* < 0.01.

**Table 3:** Conditional logistic regression of the frequency of receiving an influenza vaccination.

Variable		*N*	No. of Pneumonia	Crude OR	95% C.I.	Adjusted OR ^†^	95% C.I.
Vaccination							
1 yr	2 yr						
No	No	9882	5006	1		1	
No	Yes	1282	665	1.03	0.91–1.17	0.98	0.86–1.12
Yes	No	1817	908	0.96	0.86–1.07	0.87 *	0.78–0.98
Yes	Yes	2149	986	0.79 **	0.71–0.88	0.75 **	0.67–0.85
Vaccination							
Never		9243	4685	1		1	
Three consecutive years		1096	475	0.64 **	0.53–0.77	0.56 **	0.45–0.69

^†^ Adjusted for hypertension, diabetes, cerebrovascular disease, renal disease, liver disease, ischemic heart disease, dementia, alcohol-related disorder, and malignancy. 1 yr: receive influenza vaccine in the first year before the pneumonia diagnosis. 2 yr: receive influenza vaccine in 1 to 2 years before the pneumonia diagnosis. * *p* < 0.05, ** *p* < 0.01.

**Table 4:** Subgroup analysis of the conditional logistic regression between vaccination and non- vaccination groups.

Variable	Vaccination		Non-Vaccination		OR	95% CI
	*N*	No. of Pneumonia Event	*N*	No. of Pneumonia Event		
Age ^a^						
<40	513	271	2965	1468	1.17	0.94–1.46
40–65	219	143	2547	1240	1.69 **	1.23–2.33
≥65	4516	2145	4370	2298	0.78 **	0.71–0.86
Gender ^b^						
Female	1869	943	4113	2048	0.99	0.87–1.13
Male	3379	1616	5769	2958	0.78 **	0.7–0.87

^a^ Adjusted for hypertension, diabetes, cerebrovascular disease, renal disease, liver disease, ischemic heart disease, and malignancy. ^b^ Adjusted for hypertension, diabetes, cerebrovascular disease, renal disease, liver disease, ischemic heart disease, dementia, alcohol-related disorder, and malignancy. ** *p* < 0.01.

## References

[1] Cheng C.W., Chien M.H., Su S.C., Yang S.F. (2013). New markers in pneumonia. Clin. Chim. Acta Int. J. Clin. Chem..

[2] Anand N., Kollef M.H. (2009). The alphabet soup of pneumonia: CAP, HAP, HCAP, NHAP, and VAP. Semin. Respir. Crit. Care Med..

[3] American Thoracic Society, & Infectious Diseases Society of America (2005). Guidelines for the management of adults with hospital-acquired, ventilator-associated, and healthcare-associated pneumonia. Am. J. Respir. Crit. Care Med..

[4] Jereb M., Kotar T. (2006). Usefulness of procalcitonin to differentiate typical from atypical community-acquired pneumonia. Wien. Klin. Wochenschr..

[5] Chang G.M., Tung Y.C. (2012). Factors associated with pneumonia outcomes: A nationwide population-based study over the 1997–2008 period. J. Gen. Intern. Med..

[6] Chiu C.Y., Chen C.J., Wong K.S., Tsai M.H., Chiu C.H., Huang Y.C. (2015). Impact of bacterial and viral coinfection on mycoplasmal pneumonia in childhood community-acquired pneumonia. J. Microbiol. Immunol. Infect. = Wei Mian Yu Gan Ran Za Zhi.

[7] Cordoba E., Maduro G., Huynh M., Varma J.K., Vora N.M. (2018). Deaths from pneumonia-New York City, 1999–2015. Open Forum Infect. Dis..

[8] Huang C.Y., Chang L., Liu C.C., Huang Y.C., Chang L.Y., Huang Y.C., Chiu N.C., Lin H.C., Ho Y.H., Chi H. (2015). Risk factors of progressive community-acquired pneumonia in hospitalized children: A prospective study. J. Microbiol. Immunol. Infect. = Wei Mian Yu Gan Ran Za Zhi.

[9] Tessmer A., Welte T., Schmidt-Ott R., Eberle S., Barten G., Suttorp N., Schaberg T. (2011). Influenza vaccination is associated with reduced severity of community-acquired pneumonia. Eur. Respir. J..

[10] Shinjoh M., Sugaya N., Yamaguchi Y., Iibuchi N., Kamimaki I., Goto A., Kobayashi H., Kobayashi Y., Shibata M., Tamaoka S. (2018). Inactivated influenza vaccine effectiveness and an analysis of repeated vaccination for children during the 2016/17 season. Vaccine.

[11] Zhang X., Zhang J., Chen L., Feng L., Yu H., Zhao G., Zhang T. (2017). Pneumonia and influenza hospitalizations among children under 5 years of age in Suzhou, China, 2005–2011. Influenza Other Respir. Viruses.

[12] Almond M.H., McAuley D.F., Wise M.P., Griffiths M.J. (2012). Influenza-related pneumonia. Clin. Med..

[13] Huang H.H., Chen S.J., Chao T.F., Liu C.J., Chen T.J., Chou P., Wang F.D. (2019). Influenza vaccination and risk of respiratory failure in patients with chronic obstructive pulmonary disease: A nationwide population-based case-cohort study. J. Microbiol. Immunol. Infect..

[14] Heo J.Y., Song J.Y., Noh J.Y., Choi M.J., Yoon J.G., Lee S.N., Cheong H.J., Kim W.J. (2018). Effects of influenza immunization on pneumonia in the elderly. Hum. Vaccines Immunother..

[15] Wang I.K., Lin C.L., Chang Y.C., Lin P.C., Liang C.C., Liu Y.L., Chang C.T., Yen T.H., Huang C.C., Sung F.C. (2013). Effectiveness of influenza vaccination in elderly diabetic patients: A retrospective cohort study. Vaccine.

[16] Clar C., Oseni Z., Flowers N., Keshtkar-Jahromi M., Rees K. (2015). Influenza vaccines for preventing cardiovascular disease. Cochrane Database Syst. Rev..

[17] Casado I., Dominguez A., Toledo D., Chamorro J., Astray J., Egurrola M., Fernandez-Sierra M.A., Martin V., Morales-Suarez-Varela M., Godoy P. (2018). Repeated influenza vaccination for preventing severe and fatal influenza infection in older adults: A multicentre case-control study. Can. Med. Assoc. J..

[18] Chang T.Y., Chao T.F., Liu C.J., Chen S.J., Chung F.P., Liao J.N., Tuan T.C., Chen T.J., Chen S.A. (2016). The association between influenza infection, vaccination, and atrial fibrillation: A nationwide case-control study. Heart Rhythm.

[19] Shih C.H., Lee Y.J., Chao P.W., Kuo S.C., Ou S.M., Huang H.M., Chen Y.T. (2018). Association between influenza vaccination and the reduced risk of acute kidney injury among older people: A nested case-control study. Eur. J. Intern. Med..

[20] Vardeny O., Udell J.A., Joseph J., Farkouh M.E., Hernandez A.F., McGeer A.J., Talbot H.K., Bhatt D.L., Cannon C.P., Goodman S.G. (2018). High-dose influenza vaccine to reduce clinical outcomes in high-risk cardiovascular patients: Rationale and design of the invested trial. Am. Heart J..

[21] Wong K., Campitelli M.A., Stukel T.A., Kwong J.C. (2012). Estimating influenza vaccine effectiveness in community-dwelling elderly patients using the instrumental variable analysis method. Arch. Intern. Med..

[22] Li C., Gubbins P.O., Chen G.J. (2015). Prior pneumococcal and influenza vaccinations and in-hospital outcomes for community-acquired pneumonia in elderly veterans. J. Hosp. Med..

[23] Demirdogen Cetinoglu E., Uzaslan E., Sayiner A., Cilli A., Kilinc O., Sakar Coskun A., Hazar A., Kokturk N., Filiz A., Polatli M. (2017). Pneumococcal and influenza vaccination status of hospitalized adults with community acquired pneumonia and the effects of vaccination on clinical presentation. Hum. Vaccines Immunother..

[24] Liu J.C., Hsu Y.P., Kao P.F., Hao W.R., Liu S.H., Lin C.F., Sung L.C., Wu S.Y. (2016). Influenza vaccination reduces dementia risk in chronic kidney disease patients: A population-based cohort study. Medicine.

[25] Cauley L.S., Vella A.T. (2015). Why is coinfection with influenza virus and bacteria so difficult to control?. Discov. Med..

[26] Joseph C., Togawa Y., Shindo N. (2013). Bacterial and viral infections associated with influenza. Influenza Other Respir. Viruses.

[27] Tsai Y.H., Hsieh M.J., Chang C.J., Wen Y.W., Hu H.C., Chao Y.N., Huang Y.C., Yang C.T., Huang C.C. (2015). The 23-valent pneumococcal polysaccharide vaccine is effective in elderly adults over 75 years old—Taiwan’s PPV vaccination program. Vaccine.

[28] Chen G.W., Shih S.R., Hsiao M.R., Chang S.C., Lin S.H., Sun C.F., Tsao K.C. (2007). Multiple genotypes of influenza b viruses cocirculated in taiwan in 2004 and 2005. J. Clin. Microbiol..

[29] Alfelali M., Khandaker G., Booy R., Rashid H. (2016). Mismatching between circulating strains and vaccine strains of influenza: Effect on hajj pilgrims from both hemispheres. Hum. Vaccines Immunother..

[30] Noh J.Y., Choi W.S., Song J.Y., Lee H.S., Lim S., Lee J., Seo Y.B., Lee J.S., Wie S.H., Jeong H.W. (2018). Significant circulation of influenza b viruses mismatching the recommended vaccine-lineage in South Korea, 2007–2014. Vaccine.

